# Observations on the Superior Efficacy of the Red Peruvian Bark, in the Cure of Agues and Other Fevers. Interspersed with Occasional Remarks on the Treatment of Other Diseases, by the Same Remedy

**Published:** 1782

**Authors:** 


III. *Obfervations on the fuperior efficacy of the Red Peruvian Bark, in the cure of agues and other fevers. Interfperfed with occafional remarks on the treatment of other difeafes, by the fame remedy. By* William Saunders, M. D. *Member of the Royal College of Phyficians in London,*

*London, and Physician to Guy's Hospital.* 8vo. Johnson, *London*, 1782. 76 pages.

WHEN it is considered that in our fleets and armies the numbers, who fall a sacrifice to the epidemic fevers of warm climates, infinitely exceed those who are destroyed by the enemy; and that in almost all the dangerous fevers which occur in our East and West India settlements, the bark is a principal remedy, the utility of the present publication must be sufficiently obvious.

In the preface to this work the ingenious author informs us he had long suspected that the Peruvian bark, in common use, was inferior in power and efficacy to that recommended by Morton and Sydenham, in whose works the medical virtues of this drug, in intermittent and other fevers, are extolled as being almost infallible. In their time, he observes, the quill bark was not mentioned; their cotemporary writers on the materia medica, particularly Dale, describe the Peruvian bark of that period, as being of a larger kind, of more compact pieces, and of the colour of the rust of iron, which marks are very expressive of the red Peruvian bark. M. de la Condamine, we are told, told (*Mem. de l'Acad. des Sciences*, 1738) was ſurprized when he was informed that the writers on pharmacy and materia medica in England had preferred the ſmall and quill bark, while the inhabitants of New Spain held the larger bark in higher eſtimation. The ſame writer, ſpeaking of the three ſpecies of quinquina, the white, the yellow, and the red, obſerves that theſe three kinds differ in their virtue only, the white having ſcarce any virtue.

As a farther proof that the red bark was early in uſe in this country, our author informs us that Mr. Springall of Thames-ſtreet, whoſe uncle was, in 1702, with Sir George Rooke at Vigo, had a quantity of this ſort of bark, which was part of the plunder brought home at that time. It was purchaſed about four years ago by Mr. Pearſon, an apothecary in Spital-ſquare, who obſerved that its decoction was much ſtronger than that of the common bark.

The taſte and flavour of the red bark, it is remarked, is more difficultly evolved, and is therefore at firſt not ſo obvious from the cloſeneſs of its texture, and from its reſinous coat being incloſed between two other layers. It is evidently heavier than any other kind of bark, and ſeems to have been prepared and dried with greater greater attention, its original appearance and form being better preſerved. Dr. Saunders thinks it probable that it may be the bark of the trunk of the tree, and he is the more confirmed in this opinion by the ideas of Dr. Withering and Dr. Fothergill, who, in their letters to him on this ſubject, have remarked that the eſſential and active parts of the oak bark, are more intire, and in larger quantity in the trunk and larger branches, than in the twigs or ſmaller branches. He thinks it probable alſo, that the ſmall and quilled bark may be procured from younger trees, not yet arrived at their full maturity; and in proof of this he quotes a paſſage from the *Encyclopedia*, where it is mentioned that Mr. Arrot, a Scotch ſurgeon, who had gathered the bark in the place where it grows, found that the ſmall curled bark, ſo much eſteemed in England, is the bark of younger trees which frequently recover the barking, while the older trees never do. This affords a ſtriking proof that the early bark introduced into Europe was of the larger kind and from the older trees, while the difficulty of procuring it has been the means of introducing a ſmaller and younger bark.

The preface is followed by an introduction, in which the author informs us, that, in the year 1779, a Spaniſh ſhip from Lima, bound to Cadiz, was taken by an Engliſh frigate and carried into Liſbon; her cargo conſiſted chiefly of this bark, a part of which was afterwards brought into England, and purchaſed by ſeveral druggiſts. Our readers will recollect that, in our Journal for November 1781, we gave ſome account of this circumſtance, and of the ſingular efficacy of this remedy in the cure of intermittents.

It ſeems that although the general title by which it was ſold was that of Quinquina, yet it was ſuppoſed by our druggiſts to be a new medicine.

The work itſelf begins with a conciſe account of the Natural Hiſtory of the Bark. Our author is perſuaded, that the bark of which he is treating is the *Cinchona Officinalis* Linn. Sp. pl. 244.

The inhabitants of Old Spain, he obſerves, always preferred the larger bark, and from the relations of travellers he is diſpoſed to think, that more attention is paid in cutting and drying the bark, which is conſumed in Spain, than what is brought to a foreign market.

The

The red bark, he adds, is very diftinguifh-able from thofe large, coarfe, woody, and fibrous maffes, that are occafionally mixed with the common Peruvian bark.

Dr. Saunders next defcribes its fenfible qualities. " The red bark," he tells us, " is in " much larger and thicker pieces than the com- " mon Peruvian bark. It evidently confifts of " three layers. The external thin, rugged, " and frequently covered with a moffy fub- " ftance, and of a reddifh-brown colour. The " middle thicker, more compact, and of a " darker colour. In this appears chiefly to re- " fide its refinous part, being extremely brittle, " and evidently containing a larger quantity " of inflammable matter than any other kind of " bark.

" The innermoft has a more woody and fi- " brous appearance, of a brighter red than " the former. The intire piece breaks in that " brittle manner defcribed by writers on the " materia medica, as a proof of the fuperior " excellence of the bark.—In reducing it to " powder, the middle layer, which feems to " contain the greateft proportion of refin, will " not give way to the peftle fo eafily as the " other layers; this fhould be particularly at-

" tended

" tended to when it is ufed in fine powder. Its " flavour is chiefly difcoverable either in pow- " der or folution, is evidently more aromatic, " and has a greater degree of bitternefs than " the common bark."

We next meet with an account of feveral experiments made by our author with a view to illuftrate its chymical and pharmaceutical hif- tory. The refults are, 1. That the red bark is more foluble than common bark, both in water and fpirit; 2. That it contains a much larger proportion of active and refinous parts; 3. That its active parts, even when greatly diluted, retain their fenfible qualities in a higher degree than the moft faturated folutions of common bark; 4. That it does not undergo the fame decompofition of its parts by boiling as the common Peruvian bark.—As a proof of the fuperior antifeptic power of the red bark, we are told, that in the month of June a large quan- tity of a decoction of it, which had been kept for five weeks in the elaboratory of Guy's Hof- pital, was equally good at the expiration of that time, as when firft prepared; while a de- coction of common bark gave evident appear- ances of a change in a few days.

We

We are next prefented with fome judicious obfervations on the general operation of bark on the human body, and on its ufe in intermittent and other fevers. The author afcribes its effects chiefly to its tonic power. He obferves that in intermittents, particularly in low and marfhy fituations, and in fuch as frequently occur in the lower parts of this metropolis, the bark cannot be given too early; the ufe of either emetics or purgatives, as preparatory, being not only unneceffary, but in fome cafes productive of more debility, and therefore to be avoided. This is agreeable to the doctrine of Cleghorn, Lind, and others.

In cafes where the acute rheumatifm in its remiffions has affumed the form of a double tertian, our author has experienced the good effects of this remedy.

Four cafes are related from his own practice, of intermittent fevers cured by the red bark, after having refifted common bark and other remedies. In three of thefe cafes he employed only a cold infufion of the bark prepared by pouring a quart of cold water on two ounces of the red bark in fine powder, and frequently fhaking the mixture for the fpace of twenty-four hours.

The work clofes with extracts of letters from Mr. Jacob junior, furgeon at Feverfham, Mr. Boys, furgeon at Sandwich, Sir W. Bifhop, knight, furgeon at Maidftone, Dr. Withering, phyfician at Birmingham, Mr. Sherwin, furgeon at Enfield, and Dr. Fothergill, phyfician in London. All thefe gentlemen concur in recommending the red bark as more efficacious and powerful than any other kind, efpecially in the cure of intermittents.

Mr. Sherwin's letter contains a curious fact of a man, who took feven half ounces of alum upon the approach of as many different fits of the ague, without any other effect than its exciting a great pain in his ftomach.

Dr. Withering obferves that it will require fome farther experience to afcertain the neceffary dofes of the red bark in intermittents. He knows fome practitioners, who have given one or two drachms of it in powder every four hours betwixt the fits; but he has never had occafion to give more than thirty or forty grains at fimilar intervals. Dr. Saunders is difpofed to conclude, that it need be employed only in half the quantity we generally recommend of the other bark. We fincerely hope that this publication may excite our merchants to obtain large fupplies of this valuable drug.

